# *Salvia miltiorrhiza* polysaccharides ameliorates *Staphylococcus aureus*-induced mastitis in rats by inhibiting activation of the NF-κB and MAPK signaling pathways

**DOI:** 10.1186/s12917-022-03312-6

**Published:** 2022-05-27

**Authors:** Di Zhang, Guozhong Jin, Wei Liu, Mengmeng Dou, Xiao Wang, Wanyu Shi, Yongzhan Bao

**Affiliations:** 1 College of Traditional Chinese Veterinary Medicine, Hebei Agricultural University, 2596, Le Kai South Street, Baoding, 071001 Hebei China; 2 Hebei Provincial Veterinary Biotechnology Innovation Center, Baoding, China

**Keywords:** *Salvia miltiorrhiza* polysaccharides, *Staphylococcus aureus*, Mastitis, NF-κB, MAPK

## Abstract

The lactation capacity of dairy cows is critical to the productivity of the animals. Mastitis is a disease that directly affects the lactation capacity of cows. *Staphylococcus aureus* (*S. aureus*) is one of the most important pathogens that causes mastitis in dairy cows. The anti-inflammatory effect of *Salvia miltiorrhiza* polysaccharides (SMPs) has been demonstrated in mice and chickens. However, the effectiveness of SMPs in preventing and treating mastitis is unclear. Therefore, the purpose of this study was to explore the protective effect and mechanism of SMPs on mastitis caused by *S. aureus*. *S. aureus* was used to induce mastitis in rats, and three doses of SMPs (87.5, 175, 350 mg/kg, BW/d) were administered as treatments. The bacterial load, histopathology, and myeloperoxidase (MPO) and N-acetyl-β-D-glucosaminidase (NAGase) activities of mammary glands were observed and measured. Cytokines, including interleukin (IL)-1β, interleukin (IL)-6, and tumor necrosis factor α (TNF-α), were examined by qRT–PCR and ELISA. Key proteins in the NF-κB and MAPK signaling pathways were analyzed by Western blotting. The results showed that SMP supplementation could significantly reduce the colonization of *S. aureus* and the recruitment of inflammatory cells in mammary glands. *S. aureus*-induced gene transcription and protein expression of IL-1β, IL-6, and TNF-α were significantly suppressed in mammary glands. In addition, the increase in NF-κB and MAPK protein phosphorylation was inhibited by SMPs. These results revealed that supplementation with SMPs protected the mammary gland of rats against damage caused by *S. aureus* and alleviated the inflammatory response. This study provides a certain experimental basis for the treatment of *S. aureus-*induced mastitis with SMPs in the future.

**Supplementary Information:**

The online version contains supplementary material available at 10.1186/s12917-022-03312-6.

## Introduction

Mastitis is defined as an inflammatory reaction caused by infection of mammary gland tissue. Mastitis is one of the most common diseases in dairy cows and is a serious disease [1] that is prevalent worldwide, severely constraining the development of the global dairy industry. This disease reduces milk production, reduces quality, and increases treatment costs [2]. Mastitis is an economically important disease and is one of the most costly diseases in the dairy industry [3]. Individual cow fitness, environmental factors, and pathogenic microorganisms are the causes of mastitis in dairy cows [4]. *S. aureus*, *Streptococcus dysgalactiae*, and *Streptococcus uberis* are the main factors associated with the occurrence of mastitis in dairy cows [5]. *S. aureus* is a significant pathogenic agent in the development of mastitis in dairy cows worldwide [6]. Mastitis caused by *S. aureus* is typically treated with antibiotics, but the therapeutic effect is not satisfactory [7]. Dairy farmworkers often increase the dose of antibiotics to cure cows with inflammation, which can lead to the development of resistance in *S. aureus*, rendering many antibiotics ineffective and causing drug residues in cattle byproducts [8], which has implications for human health. However, the search for new therapeutic alternatives that are effective in controlling and treating bovine mastitis is urgent [9].

Previous authors have conducted extensive research on the defense mechanisms of mammary tissue. Pathogen-associated molecular patterns (PAMPs) of *S. aureus* can be recognized by pattern recognition receptors (PRRs) in cells. The downstream nuclear factor κB (NF-κB) and mitogen-activated protein kinase (MAPK) signaling pathways are activated [10]. Cytokines recruit leukocytes to the site of infection, which is crucial in the early stages of inflammation [11].

Chinese herbal medicines have immunomodulatory effects [12]. Low toxicity, low residue and no drug resistance are characteristics of these treatment. Many dairy farms in China are using herbal remedies to treat mastitis in cows [13]. *Salvia miltiorrhiza* is a traditional Chinese herbal medicine that can activate blood flow and remove the blood stasis effect caused by dextran and is the main ingredient in prescription drugs for treating mastitis in cows [14]. *Salvia miltiorrhiza* extract includes tanshinone and SMPs. One study showed that TBF had an obvious effect against acute inflammation and chronic inflammation in mice and suppressed pathogenic bacteria in vitro and in vivo [15]. Yang found that tanshinone I or tanshinone IIA/B significantly inhibited activation of the NF-κB signaling pathway after LPS stimulation of mammary epithelial cells in mice, thereby attenuating the inflammatory response [16]. SMPs is another extract of *Salvia miltiorrhiza* that has anti-inflammatory effects. SMPs can improve the liver index, spleen index and thymus index, reduce the serum levels of alanine aminotransferase, aspartate aminotransferase and nitric oxide, restore liver levels of TNF-α and IL-1β, and ameliorate immunological liver injury induced by Bacille-Calmette-Guerin (BCG) and lipopolysaccharide (LPS) in mice [17]. Geng found that SMPs may alleviate florfenicol-induced liver injury in broilers by inhibiting activation of the PPAR and MAPK signaling pathways [18]. However, the therapeutic effect and mechanism of SMPs on mastitis in dairy cows have not been reported. In this study, a rat mastitis model was established. Pathological changes in the mammary gland were observed by histopathology. qRT–PCR, ELISA and Western blotting were used to analyze inflammatory factors. Various experimental techniques were used to verify whether SMPs could exert anti-inflammatory effects by regulating the activation of the NF-κB and MAPK signaling pathways. This study is valuable for the development of SMPs as immunomodulatory drugs to treat animals.

## Results

### Effects of SMPs on bacterial load in mammary tissue

**Fig. 1:**
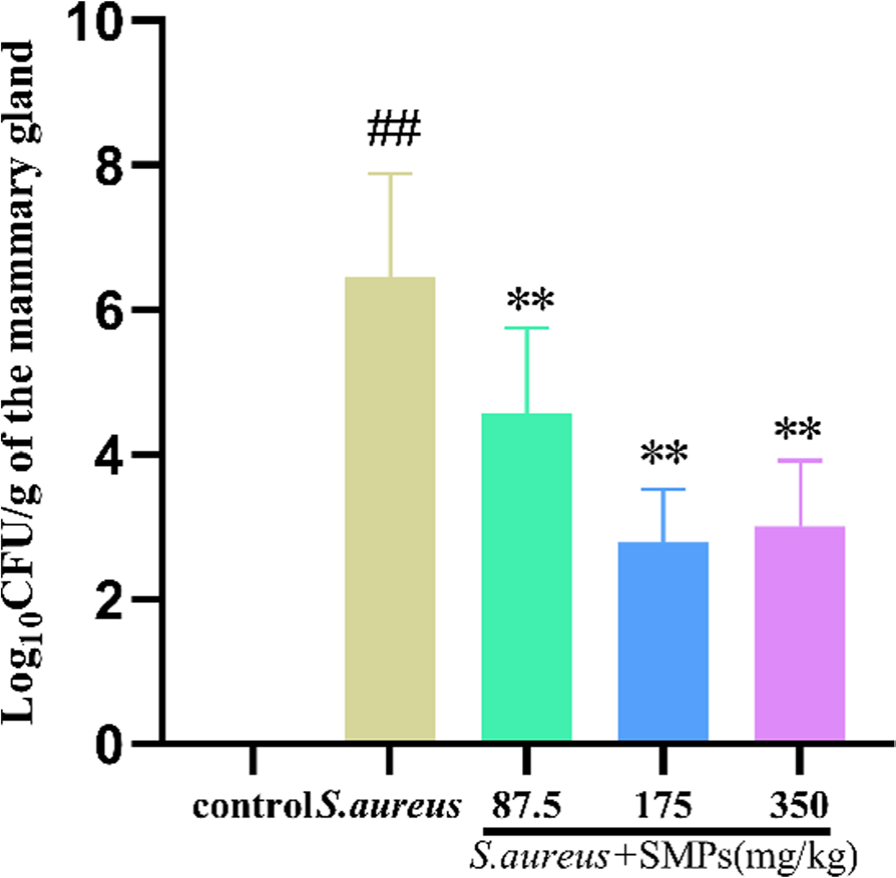
*S. aureus* load in mammary tissue. The data are expressed as the mean Log_10_CFU/g ± SD of mammary glands (*n* = 8). *##p* < 0.01 vs. control. **p < 0.01 vs. *S. aureus*

As shown in Fig. 1, mammary tissue was collected 24 h after infection, and *S. aureus* colonization was analyzed. No bacterial colonization was observed in the control group. Compared with the *S. aureus* group, the SMP treatment groups had a significantly reduced bacterial loads in mammary tissue (*P* < 0.01). There was no significant difference between the medium- and high-dose groups (*P* > 0.05).

### Effect of SMPs on histopathological changes

**Fig. 2:**
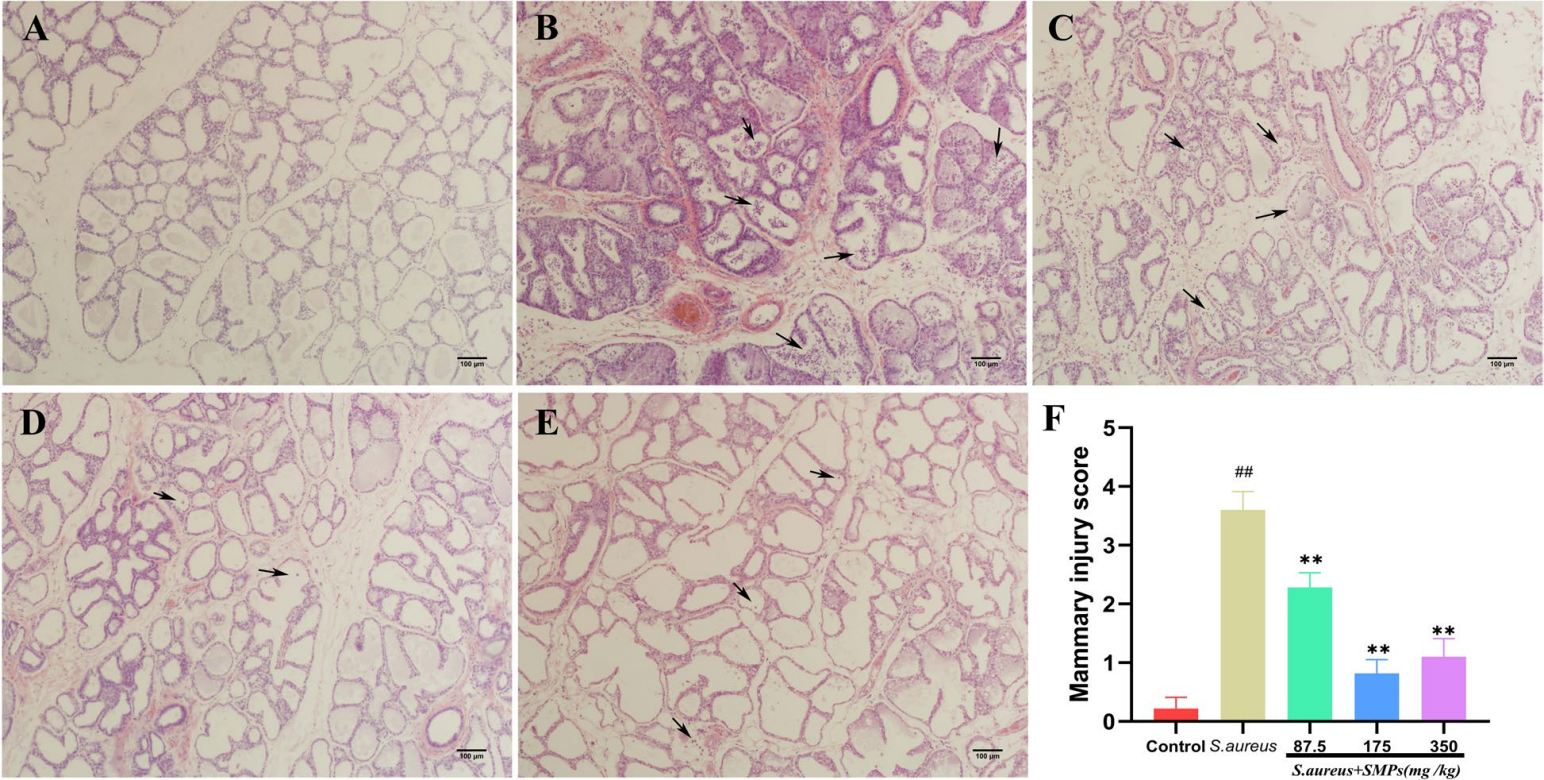
Histopathological changes in the mammary tissue of rats in each group. (H&E 100×). **A** Control group. **B** *S. aureus* group. **C**-**E** *S. aureus* + SMP groups (87.5, 175, 350 mg/kg). **F** Mammary injury score. *##p* < 0.01 vs. control. **p < 0.01 vs. *S. aureus*. The black arrows represent the presence of inflammatory cell infiltrates and ruptured mammary epithelial cells

As shown in Fig. 2, the control group had an intact glandular follicular structure in mammary tissue, milk filling, and no histopathological changes (Fig. 2A). However, significant inflammatory changes were observed in the mammary tissue of the *S. aureus* group, and inflammatory cells such as neutrophils and macrophages infiltrated the acinar chamber, mammary ducts, perivascular and connective tissues (Fig. 2B). Inflammatory cell infiltration and mammary tissue damage were significantly reduced in each SMP group (Fig. 2C-E). Pathological histological scoring was performed, and the *S. aureus* group scored significantly higher than the control group (*p* < 0.01). After pretreatment with SMPs, the score of the SMP group was significantly lower than that of the *S. aureus* group (*p* < 0.01) (Fig. 2F).

### Effect of SMPs on MPO expression levels in mammary tissues

**Fig. 3:**
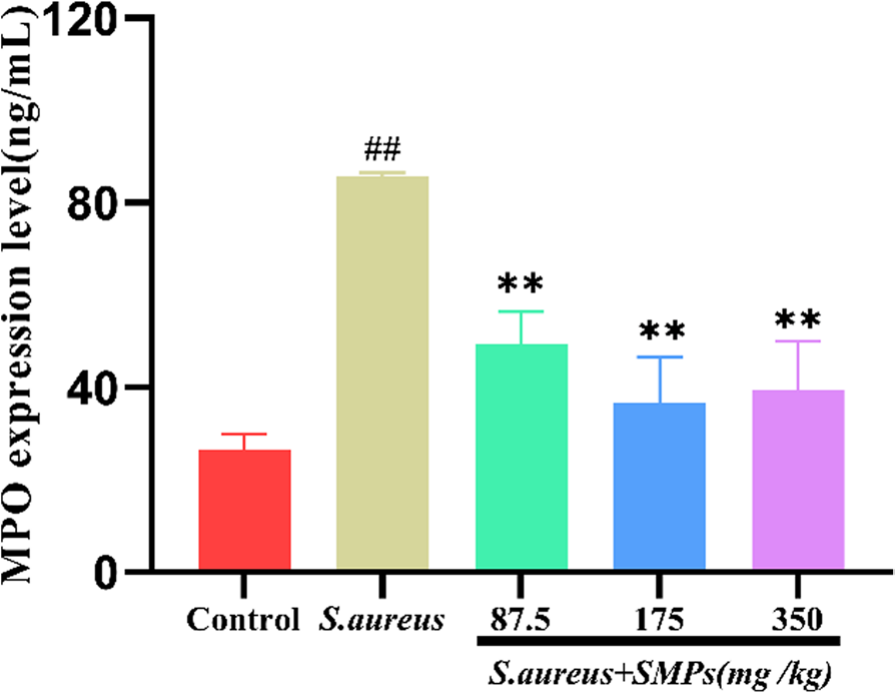
MPO expression levels in mammary tissues. The data are the mean ± SD (*n* = 8), *##p* < 0.01 vs. control. **p < 0.01 vs. *S. aureus*

As shown in Fig. 3, the MPO expression level was higher in the *S. aureus* group than in the control group (*P* < 0.01). Compared with that in the *S. aureus* group, supplementation with SMPs effectively inhibited MPO activity, and the most pronounced effect was observed in the medium- and high-dose groups, in which MPO expression was significantly reduced (*P* < 0.01). The difference was not significant (*P* > 0.05) between the medium- and high-dose groups.

### Effect of SMPs on NAGase expression levels in mammary tissues

**Fig. 4:**
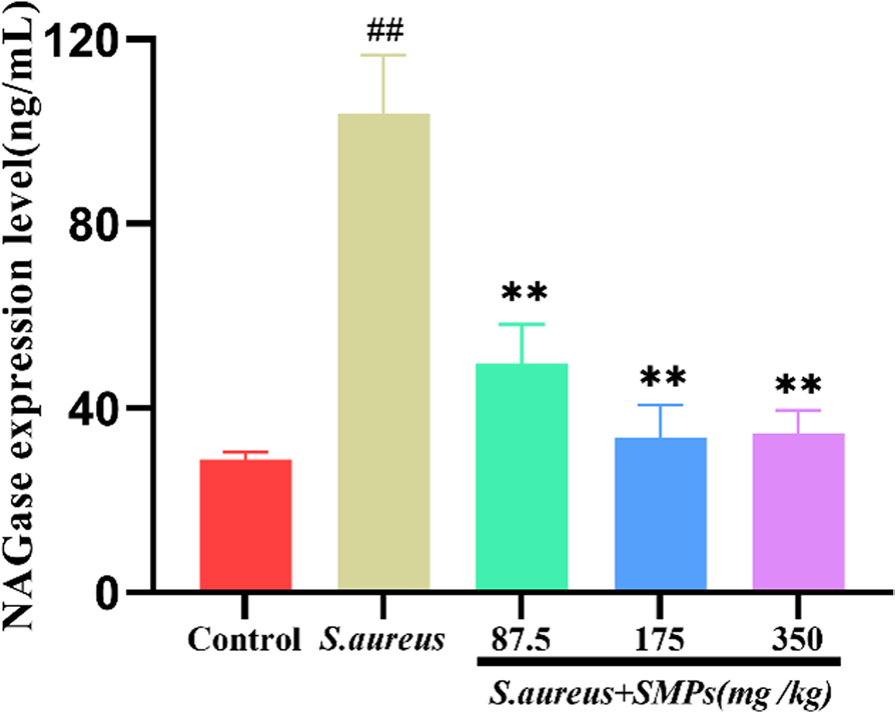
NAGase expression levels in mammary tissues. The data are the mean ± SD (*n* = 8), *##p* < 0.01 vs. control. **p < 0.01 vs. *S. aureus*

As shown in Fig. 4, the NAGase expression level was higher in the *S. aureus* group than in the control group (*P* < 0.01). Compared to that in the *S. aureus* group, supplementation with SMPs effectively inhibited NAGase activity. The decline was more pronounced in the middle- and high-dose groups than in the low-dose group (*P* < 0.01). The effect was not significant (*P* > 0.05) between the medium- and high-dose groups.

### Effect of SMPs on inflammatory cytokine mRNA expression

**Fig. 5:**
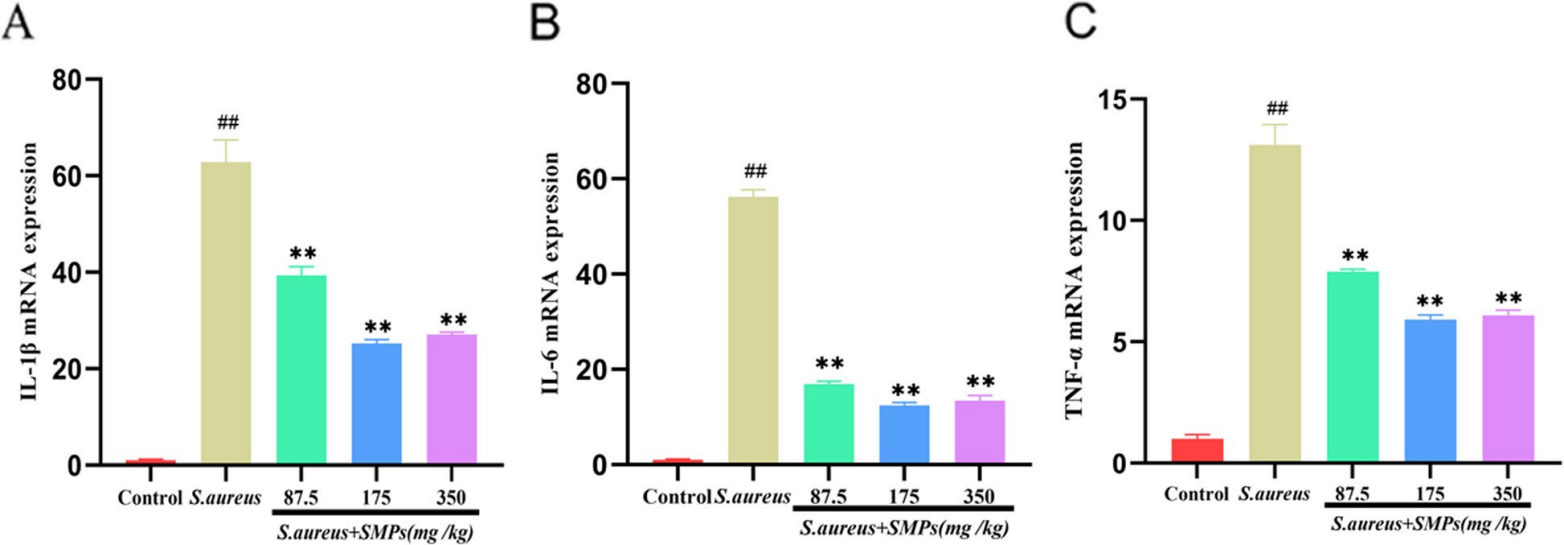
The effects of SMPs on the mRNA expression levels of IL-1β (A), IL-6 (B) and TNF-α (C). The data are the mean ± SD, *##p* < 0.01 vs. control. **p < 0.01 vs. *S. aureus*

The mRNA expression levels of IL-1β, IL-6, and TNF-α in mammary tissues were measured using qRT–PCR. As shown in Fig. 5, the mRNA expression levels of IL-1β, IL-6 and TNF-α were higher in the *S. aureus* group than in the control group (*P* < 0.01). The expression levels of IL-1β, IL-6 and TNF-α were lower in the SMP pretreatment group than in the *S. aureus* group (*P* < 0.01). The medium dose group had more obvious improvements than the other groups.

### Effect of SMPs on inflammatory cytokine expression levels

**Fig. 6:**
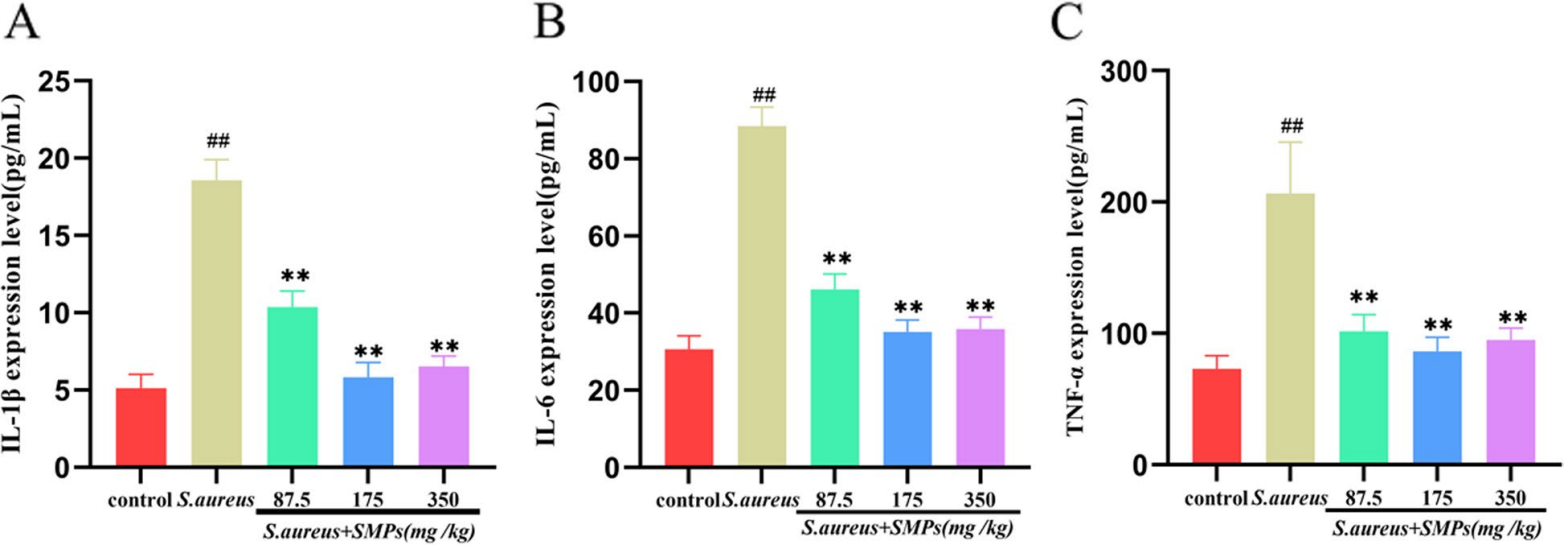
The effects of SMPs on the expression of the inflammatory cytokines IL-1β (**A**), IL-6 (**B**) and TNF-α (**C**). The data are the mean ± SD, *##p* < 0.01 vs. control. **p < 0.01 vs. *S. aureus*

IL-1β, IL-6, and TNF-α expression levels in mammary tissues were examined by ELISA. As shown in Fig. 6, the expression levels of IL-1β, IL-6 and TNF-α were higher in the *S. aureus* group than in the control group (*P* < 0.01). The expression levels of IL-1β, IL-6 and TNF-α were lower in the SMP pretreatment group than in the *S. aureus* group (*P* < 0.01). The medium dose group had more obvious improvements than the other groups. The ELISA and qRT–PCR results were consistent.

### Effect of SMPs on NF-κB signaling pathway activation

**Fig. 7:**
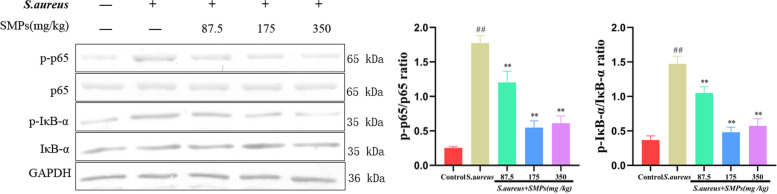
Effects of SMPs on the NF-κB signaling pathway in mammary tissues. Immunoblots and acquisition of intensity from the respective blots. The protein expression was normalized by the respective abundance of GAPDH. Full-length blots are presented in Additional file. All results are expressed as the means ± SD (*n* = 3). *##p* < 0.01 vs. control. **p < 0.01 vs. *S. aureus*

As shown in Fig. 7, the phosphorylation levels of p65 and IκB-α were significantly elevated in the *S. aureus* group compared to the control group (*P* < 0.01). The phosphorylation of p65 and IκB-α was significantly inhibited by different doses of SMP (*P* < 0.01), and the best effect was observed in the medium-dose group.

### Effect of SMPs on MAPK signaling pathway activation

**Fig. 8:**
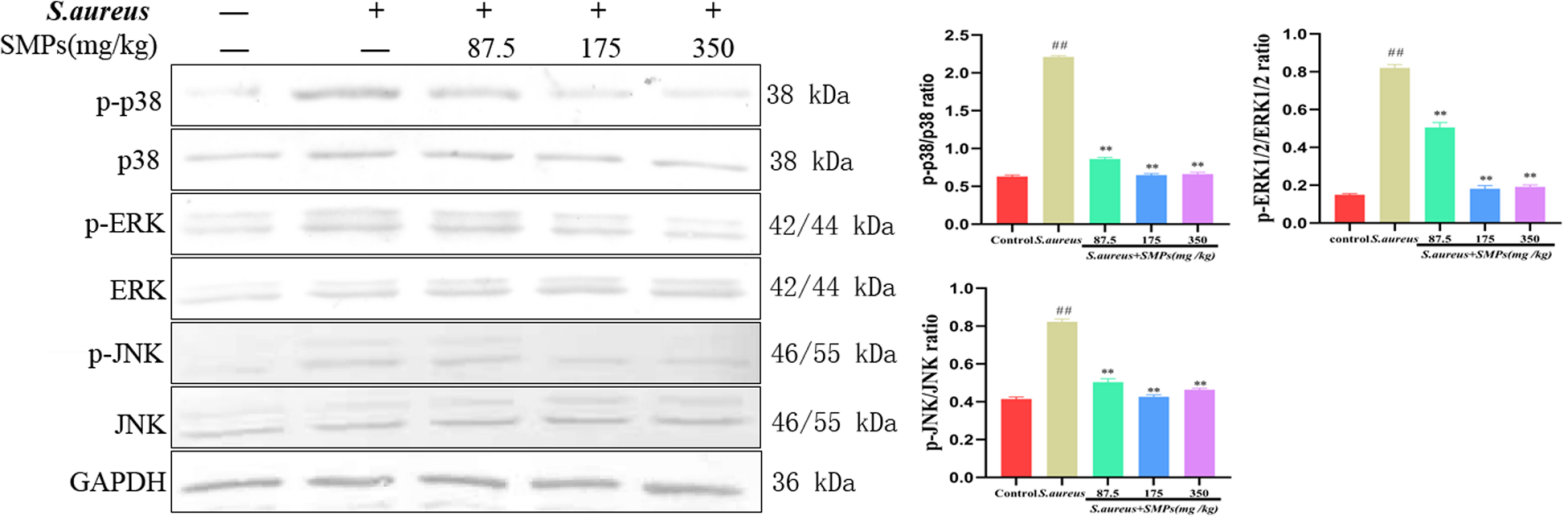
The effects of SMPs on the MAPK signaling pathway in mammary tissues. Immunoblots and acquisition of intensity from the respective blots. The protein expression was normalized by the respective abundance of GAPDH. Full-length blots are presented in Additional file. All results are expressed as the means ± SD (*n* = 3). *##p* < 0.01 vs. control. **p < 0.01 vs. *S. aureus*

As shown in Fig. 8, the phosphorylation levels of p38, ERK, and JNK were significantly increased in the *S. aureus* group compared to the control group (*P* < 0.01). The phosphorylation of p38, ERK, and JNK was significantly inhibited by different doses of SMP (*P* < 0.01), and the best effect was observed in the medium-dose group.

## Discussion

Mastitis is one of the most serious diseases in dairy cows and severely restricts the development of the dairy industry [19]. *S. aureus* is one of the main pathogens associated with mastitis in dairy cows, and *S. aureus* is easily resistant to antibiotics, making treatment difficult. Dairy farm employees often use large amounts of antibiotics in their daily treatments, which causes antibiotics to accumulate in the animals. This accumulation leads to health risks for human. Therefore, it is urgent to find drugs with low toxicity and low residue to treat mastitis in dairy cows. SMPs could inhibit activation of the NF-κB signaling pathway and thus attenuate liver injury caused by lipopolysaccharide (LPS) in mice [20]. Han found that administered 5 g/L SMPs to chicks for five consecutive days beginning on the first day after hatching could effectively increase the blood antibody titer and immunoglobulin levels of broilers [21]. However, it is not clear whether SMPs can relieve mastitis caused by *S. aureus*. The rat, as a standard test animal, can perfectly simulate bovine mastitis, which is also very common in scientific research. Therefore, the present study was conducted to evaluate the anti-inflammatory effect of SMPs on an *S. aureus*-induced mastitis rat model.

During bacterial infections, reducing bacterial colonization is essential to controlling the infection [22]. In this study, we found that mammary tissue was heavily colonized with bacteria, which was similar to the findings of previous studies [23]. Supplementation with SMPs effectively reduced bacterial colonization. This result suggests that SMPs enhance the phagocytic ability of macrophages and improve the immune response in the body [24]. Histopathological examination showed many infiltrating inflammatory cells and necrotic detached mammary epithelial cells in severely damaged mammary tissue after *S. aureus* infection, which is consistent with previous studies [25]. However, supplementation with SMPs reduced inflammatory cell infiltration and mitigated tissue damage. MPO is a marker of neutrophil infiltration [26]. MPO was increased in mammary tissue after *S. aureus* infection [27], but MPO activity was significantly inhibited by supplementation with SMPs. Therefore, supplementation with SMPs can reduce inflammatory cell infiltration to alleviate the inflammatory response. It was found that NAGase activity was significantly increased in rat mammary tissue after infection with *S. aureus* [28]. NAGase is mainly derived from mammary epithelial cells, and NAGase is released when mammary epithelial cells are destroyed. This factor is a marker of the degree of mammary epithelial cell damage [29]. The level of NAGase in mammary tissue is directly proportional to the severity of mastitis. In this study, we found that NAGase activity in mammary tissue was significantly reduced after supplementation with SMPs. This result suggested that SMPs reduced the infiltration of inflammatory cells, attenuated the inflammatory response, and prevented the destruction of mammary epithelial cells.

The proinflammatory cytokines IL-1β, IL-6, and TNF-α are important in mastitis. IL-1β can be produced by a variety of cells, and it can be involved in the recruitment of neutrophils to control *S. aureus* infection [30]. IL-6 is a proinflammatory cytokine that is increased rapidly in response to acute inflammation and is proportional to the degree of injury and infection. TNF-α is a proinflammatory cytokine released by macrophages that plays an important role in regulating the early inflammatory response. Adequate production of cytokines following bacterial infection is vital for host immune defense. However, continued production of cytokines can also lead to cellular damage, which can lead to mastitis. Our study found a significant decrease in the expression levels of IL-1β, IL-6 and TNF-α after supplementation with SMPs. This suggests that SMPs can inhibit the expression of pro-inflammatory cytokines. Gilbert found no change in the protein levels of IL-1β and TNF-α after *S. aureus* infection of bovine mammary epithelial cells [31]. Wellnitz found that TNF-α and IL-1β mRNA expression showed an increase in milk cells and that LPS was a stronger inducer than LTA [32]. Bannerman found a significant increase in IL-1β levels in milk after *S. aureus* infection, while TNF-α levels did not change, so the authors suggested that the limited cytokine response to *S. aureus* may contribute to the well-known ability of the bacterium to establish chronic intramammary infection [33]. There is no doubt that *S. aureus* causes persistent infections. However, whether *S. aureus* infection affects cytokine expression is a question that needs to be explored. We examined the expression of IL-1β, IL-6 and TNF-α after infection with *S. aureus*. It was found that the expression levels of IL-1β, IL-6, and TNF-α were significantly increased after *S. aureus* infection in rat mammary glands. For the two different conclusions we reviewed the literature and found that *S. aureus* was able to cause an increase in pro-inflammatory cytokines in rat and mouse mastitis [28, 34–37]. We consider that the reason for the two different results may be related to differences in species and sample collection. Cytokine levels may be more easily detected in mammary tissue compared to milk. Whether there are differences between bovine and rat mastitis caused by *S. aureus*? This reminds of the shortcomings in our study and there it is necessary to compare the differences between mastitis induced by *S. aureus* in cows, rats, and mice in future studies.

NF-κB is widely present in various eukaryotic cells and can regulate the transcription of various inflammation-related factors [38]. Moreover, the NF-κB signaling pathway can be activated by various cytokines, including IL-1β, IL-6, and TNF-α [39]. NF-κB contains the subunits p50/p65, which form a heterodimer in the cell [40]. Under normal physiological conditions, IκB and NF-κB interact so that NF-κB is present in an inactive state [41]. External stimuli induce the ubiquitination of IκB-α in cells, thereby causing the degradation of IκB-α by proteases [42]. Correspondingly, NF-κB is activated. The p65 subunit enters the nucleus, and cytokines are transcribed [43]. Günther et al. found that *S. aureus* will slowly elicit a much weaker inflammation and immune response, frequently resulting in chronic infections. And failure to activating IκB/NF-κB signaling [44]. Schukken et al. reported that *S. aureus* impaired NF-κB activation in MECs resulted in a very low cytokine expression as measured by mRNA quantity [45]. But Liu found that *S. aureus* significantly increased the phosphorylation of IκB-α and p65 in mammary tissue [23]. The present study obtained similar results, suggesting that NF-κB is involved in the inflammatory response. We performed pre-experiments before the experiment. Mammary tissues of rats were infected with different concentrations of *S. aureus*. The phosphorylation level of NF-κB p65 was examined by Western blot. The experimental results showed that the phosphorylation levels increased with increased bacterial concentrations. Therefore, we consider that the concentration of bacterial infection is an important factor affecting the inflammatory response. Wang found that SMPs could alleviate lipopolysaccharide/d-galactosamine-induced liver injury in mice by inhibiting the activation of the NF-κB signaling pathway [46]. In the present study, supplementation with SMPs significantly inhibited the phosphorylation of IκB-α and p65 in rat mammary glands after infection with *S. aureus*. Combined with the inhibitory effect of SMPs on the mRNA expression of inflammatory cytokines, SMPs can negatively regulate the NF-κB signaling pathway to attenuate the inflammatory response.

The MAPK signaling pathway also has an important role in the inflammatory response. MAPK is involved in several cellular inflammatory responses, and it is closely associated with cell proliferation, differentiation, and apoptosis [47]. MAPK, similar to NF-κB, can positively regulate the transcription of cytokines [48]. MAPK consists of four major signaling pathways: p38MAPK, extracellular signal-related kinase (ERK), Jun N-terminal kinase (JNK), and big MAPK (BMK) [49]. The most widely studied kinases are p38, ERK, and JNK. These factors are phosphorylated by MKKK and MKK kinase cascades [50]. The p38, ERK, and JNK pathways are involved in the inflammatory response by regulating the expression of inflammatory cytokines [51]. Zhang showed that *S. aureus* could induce the phosphorylation of p38, ERK, and JNK in mouse mammary tissue [37]. In this study, the same results were obtained as in the previous study. Significant upregulation of p38, ERK, and JNK phosphorylation after *S. aureus* infection was observed. MAPK is a classic inflammatory pathway, and this study showed that SMPs could control the inflammatory response by inhibiting MAPK signaling pathway activation. Supplementation with SMPs significantly downregulated the levels of *S. aureus*-induced p38, ERK, and JNK phosphorylation. SMPs could alleviate mastitis in rats by regulating the MAPK signaling pathway.

## Conclusions

Supplementation with SMPs can reduce bacterial colonization, inhibit MPO and NAGase activity, and reduce the infiltration of inflammatory cells, thus protecting mammary tissue of rats from damage caused by excessive inflammatory responses. The anti-inflammatory effect of SMPs on *S. aureus*-induced mastitis in rats may be related to suppressing inflammatory cytokine gene expression by inhibiting the activation of the NF-κB and MAPK signaling pathways. It found that the immune response of mammary glands during *S. aureus* mastitis in dairy cows and rats differs significantly. However, this study has some theoretical guidance for the alleviative effect of SMPs on *S. aureus*-induced mastitis. Then, we will choose dairy cows as test animals to study the therapeutic effect of SMPs on *S. aureus*-induced mastitis in the future.

## Materials and methods

### Reagents

The isolation and purification of SMPs from the roots of *Salvia miltiorrhiza* have been reported in our previous work [52]. *S. aureus* was isolated and identified from dairy cows with mastitis and stored in the Preventive Veterinary Laboratory of Hebei Agricultural University. Myeloperoxidase assay kits (01/2021), an N-acetyl-β-D-glucosaminidase assay kit (01/2021), an interleukin-1β kit (01/2021), an interleukin-6 kit (01/2021), and a tumor necrosis factor-alpha kit (01/2021) were obtained from Mlbio Technology Co., Ltd. (Shanghai, China). Primary antibodies against IκB-α (BA04157935), p-IκB-α (AB01305600), p65 (BJ10155935), p-p65 (AH04238120), p38 (BA03091093), p-p38 (BJ14306511), ERK1/2 (BA13042500), p-ERK1/2 (BJ11066246), JNK (AB00086503), p-JNK (BJ08063309), and Gapdh (AH10017731) were provided by Bioss Technology Co., Ltd. (Beijing, China).

### Animal treatment

Thirty 8-week-old pregnant Wistar rats (weight: 190–210 g) were obtained from SPF Biotechnology Co. Ltd. (Beijing, China). The rats were reared in standard plastic cages with a 12-hour light/dark cycle. Room temperature was maintained at 23 ± 2 °C, and the relative humidity was 55 ± 5%. Standard chow and water were freely available. The rats were randomly divided into the following five groups (*n* = 8): (A) control group, (B) *S. aureus* group, and (C-E) *S. aureus* + SMPs low-, medium-, and high-dose groups (87.5, 175, 350 mg/kg body weight/day, dissolved in distilled water). The SMP dose group underwent seven consecutive days of gavage starting from the first day of delivery. On the 8th day after delivery, the rats were anesthetized with ether. The rats were washed and disinfected. L4 and R4 mammary glands in Groups B-E were injected with 100 μL of a *S. aureus* suspension (2 × 10^8^ CFU/ml) using a microinjector through the nipple catheter. The rats in the control group were injected with the same amount of physiological saline. All rats were euthanized by cervical dislocation after ether anesthesia 24 h after infection. The mammary tissue was collected aseptically and divided into three parts. One part was used to analyze bacterial load, one part was fixed in 4% formaldehyde, and the rest was stored at − 80 °C.

### Bacterial load in the mammary glands

A 10-fold serial dilution method was used to calculate the number of colonies of *S. aureus* in mammary tissues to evaluate bacterial colonization in mammary tissues. Using a tissue homogenizer, 0.1 g of the mammary gland was homogenized in 1 ml of phosphate-buffered saline (PBS). The homogenate was diluted by a 10-fold serial dilution method. Then, 100 μL of homogenate was pipetted onto LB agar plates and incubated at 37 °C for 12 h. The number of *S. aureus* colonies was calculated. SPSS one-way ANOVA was used to analyze the differences between groups.

### Histopathological observations

The mammary tissue was trimmed into small pieces and fixed with 10% formalin. Mammary tissue was dehydrated in gradient alcohol, followed by clearing using xylene and paraffin-embedding. The prepared paraffin sections were stained using hematoxylin and eosin (H&E). Histopathological changes were imaged and analyzed under a light microscope. The extent of damage to mammary tissue was assessed using a scoring system based on a previous study [53]. Three analysts scored the sections. The degree of damage to the mammary tissue was used as the basis for scoring. Each section was divided into five fields of view. A score of 0–4 represents no impairment, mild impairment, moderate impairment, severe impairment, and very severe impairment, respectively.

### Myeloperoxidase expression levels in mammary glands

Myeloperoxidase (MPO) expression levels in mammary tissue were assayed using a commercial kit. Specifically, mammary tissue was mixed with PBS 1:9 (w/v). The tissue was homogenized using a homogenizer, and the supernatant was obtained by centrifugation at 3000 r/min for 20 min. The enzymatic activity was calculated by measuring the absorbance at 450 nm.

### N-acetyl-β-D- glucosaminidase expression level in mammary glands

A commercial kit was used to determine N-acetyl-β-D- glucosaminidase (NAGase) activity in mammary tissue. Specifically, mammary tissue was mixed with PBS 1:9 (w/v). The tissue was homogenized using a homogenizer, and the supernatant was obtained by centrifugation at 3000 r/min for 20 minutes. The enzymatic activity was calculated by measuring the absorbance at 450 nm.

### Determination of inflammatory cytokine gene transcription by qRT–PCR

Total RNA was extracted from mammary tissue using a Total RNA Extraction Kit. (Shanghai Promega Biotech Co., Ltd., China). The concentration and purity of RNA were measured using a JENWAY GENOVA NANO spectrophotometer (Bibby Scientific Co., Ltd., UK). RNA samples with an A260/280 ratio of 1.90–2.0 were selected. cDNA synthesis was performed using the PrimeScript RT reagent kit with gDNA Eraser (TaKaRa Biotech Co., Ltd., China). Based on the gene sequences provided by NCBI, TaKaRa Biotech Co., Ltd. was commissioned to design and synthesize the primers. The primer sequences are shown in Table 1. qRT-PCR was performed using a Roche LightCycler96 real-time fluorescent quantitative PCR instrument. Relative mRNA expression levels were calculated using the 2^-ΔΔCt^ method with β-actin as the reference gene.

**Table 1:** Primer sequences for qRT-PCR

Gene		Primer	Length (bp)
IL-1β	sense	5′-AAAAATGCCTCGTGCTGTCT-3′	118
	antisense	5′- TCGTTGCTTGTCTCTCCTTG-3′	
IL-6	sense	5′- AGTTGCCTTCTTGGGACTGA-3′	102
	antisense	5′- ACTGGTCTGTTGTGGGTGGT-3′	
TNF-α	sense	5′- GTCGTAGCAAACCACCAAGC-3′	147
	antisense	5′-GAAGAGAACCTGGGAGTAGATAAGG-3′	
β-Actin	sense	5′- GCTCTCTTCCAGCCTTCCTT-3′	101
	antisense	5′- AGGTCTTTACGGATGTCAACG-3′	

### Determination of inflammatory cytokines by ELISA

The expression levels of cytokines in mammary tissues were measured using ELISA. The mammary tissue was homogenized with PBS at a ratio of 1:9 (w/v) and then centrifuged at 2000 r/min for 20 min at 4 °C to obtain the supernatant. Interleukin (IL)-1β, IL-6, and tumor necrosis factor (TNF)-α levels were measured using enzyme-linked immunosorbent assay (Mlbio Technology Co., Ltd., Shanghai, China). The OD value of each well was measured on a microplate reader at a wavelength of 450 nm. The standard curve was plotted according to the instructions. To prepare the corresponding curve, the OD value of the absorbance was the vertical coordinate (Y), and the concentration of the corresponding substance to be measured was the horizontal coordinate (X). SPSS one-way ANOVA was used to analyze the differences between groups [54].

### Determination of key proteins by Western blotting

Mammary tissue was homogenized using RIPA lysis buffer containing protease and phosphatase inhibitors, and the supernatant was obtained after centrifugation at 12,000 rpm for 17 minutes at 4 °C. Protein concentrations were measured using a BCA Protein Assay Kit (Shanghai Beyotime Biotechnology Co., Ltd., China). The protein concentrations in the samples were adjusted to be consistent. The proteins in the samples were separated in 8–12% SDS-polyacrylamide gels and transferred to nitrocellulose membranes. Subsequently, skim milk powder was diluted to a concentration of 5% using TBST. The membrane was blocked for 2 h at room temperature in 5% skim milk. The membranes were incubated with IκB-α, p-IκB-α, p65, p-p65, p38, p-p38, ERK, p-ERK, JNK, p-JNK and GAPDH primary antibodies (all diluted 1:1000 in 5% BSA) overnight at 4 °C. GAPDH was used as an internal reference. Subsequently, the membranes were incubated with secondary antibodies conjugated with horseradish peroxidase for 2 h at room temperature. The secondary antibodies (1:1000) was diluted in 5% BSA according to the manufacturer’s instructions. Finally, the blots were exposed using DAB Chromogenic kit. Bands were quantified and analyzed by Image J.

### Statistical analysis

All data were analyzed using SPSS 19.0 software (IBM, USA), and the data are expressed as the mean ± standard deviation (SD). Statistical significance was assessed by one-way analysis of variance (ANOVA) and the least significant difference (LSD) t test for differences. *##p* < 0.01 vs. control. *p < 0.05 vs. *S. aureus*. **p < 0.01 vs. *S. aureus*.

## Supplementary Information

**Additional file 1.**

## Data Availability

The datasets used and/or analyzed in the current study are available from the corresponding author upon reasonable request. The primer sequence used in this study was designed according to the gene sequence provided by the gene repository of the National Biotechnology Information Center (NCBI-GENE; https://www.ncbi.nlm.nih.gov/gene). The accession numbers of gene sequence are IL-1β (NM_031512.2), IL-6 (NM_012589.2), TNF-α (NM_012675.3), β-Actin (NM_031144.3).

## Abbreviations

DAB: 3,3 N-Diaminobenzidine Tertrahydrochloride
ERK: Extracellular signal-related kinase
H&E: Hematoxylin and eosin
IL-1β: Interleukin-1β
IL-6: Interleukin-6
JNK: Jun N-terminal kinase
LPS: Lipopolysaccharide
MAPK: Mitotic-activated protein kinase
MPO: Myeloperoxidase
NAGase: N-acetyl-β-D-glucosaminidase
NF-κB: Nuclear transcription factor κB
*S. aureus*: *Staphylococcus aureus*
SMPs: *Salvia miltiorrhiza* polysaccharides
TNF-α: Tumor necrosis factor-α
