# Supplementary figures and images for: Salvia miltiorrhiza polysaccharides ameliorates Staphylococcus aureus-induced mastitis in rats by inhibiting activation of the NF-κB and MAPK signaling pathways
Source: BMC Vet Res. 2022 May 27;18:201. doi: 10.1186/s12917-022-03312-6 (PMC9137159; doi:10.1186/s12917-022-03312-6)

**
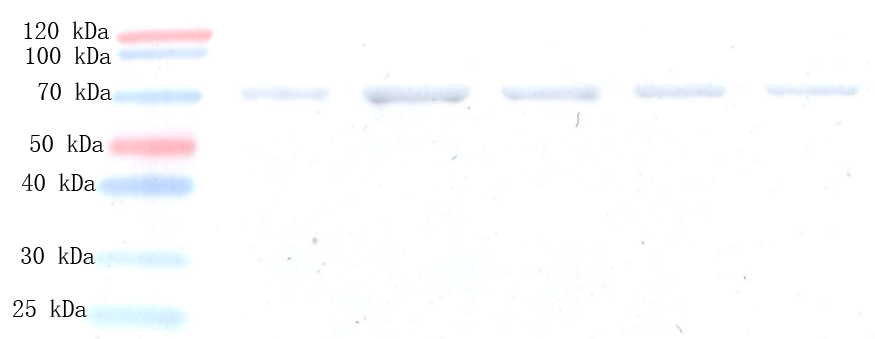
**

**p-p65**


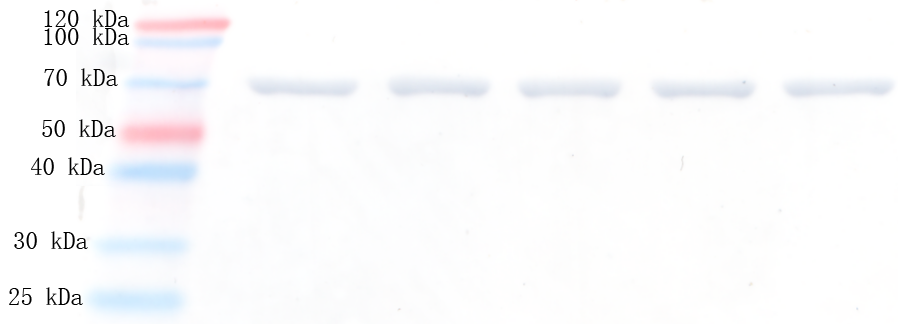


**p65**

**
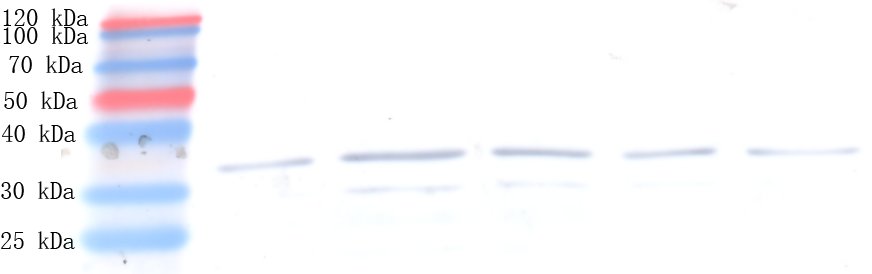
**

**p-IκB-α**

**
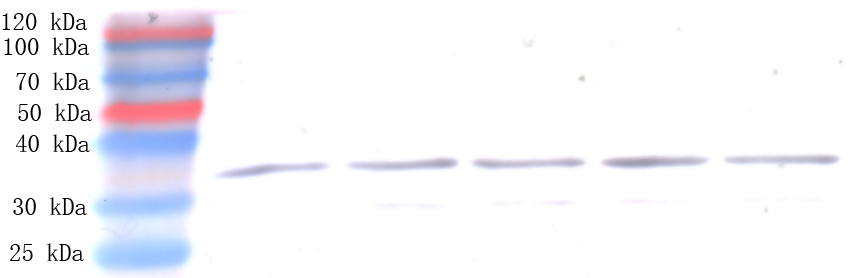
**

**IκB-α**

**
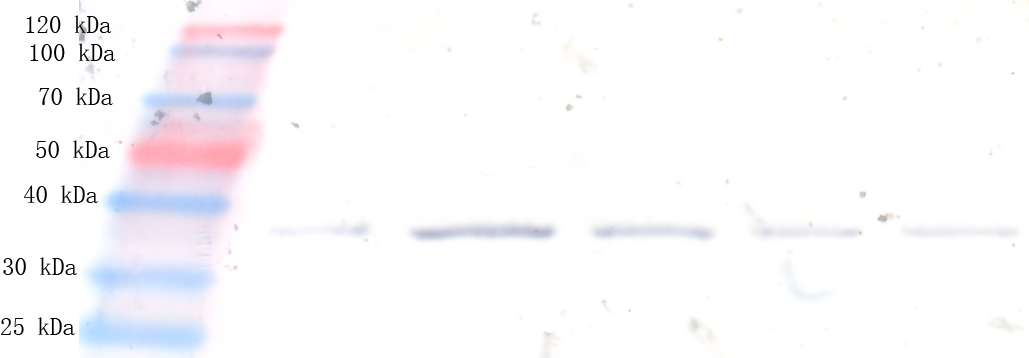
**

**p-p38**

**
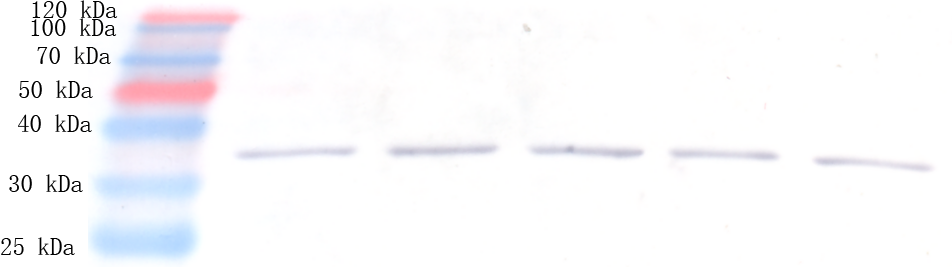
**

**P38**

**
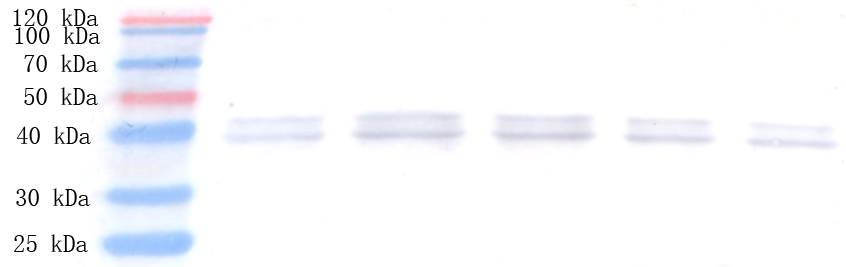
**

**p-ERK**

**
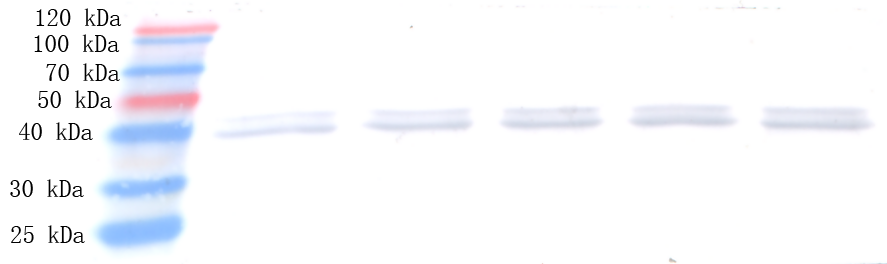
**

**ERK**

**
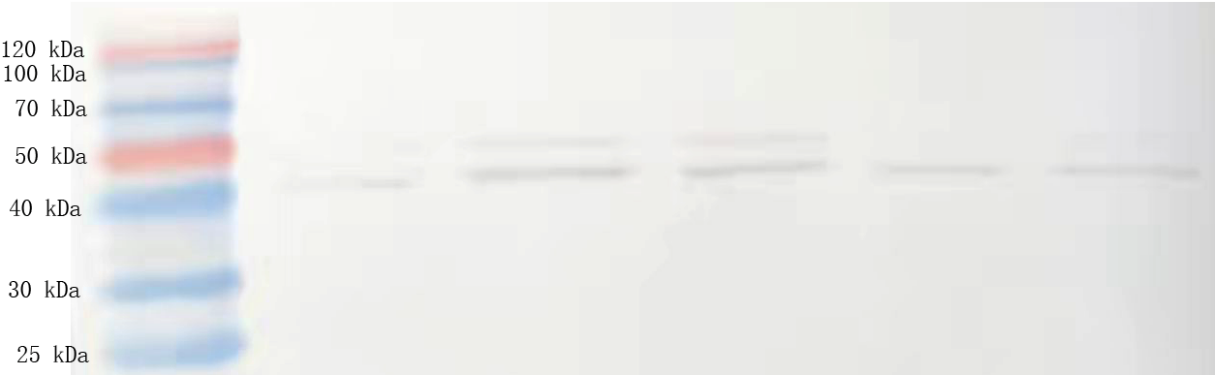
**

**P-JNK**

**
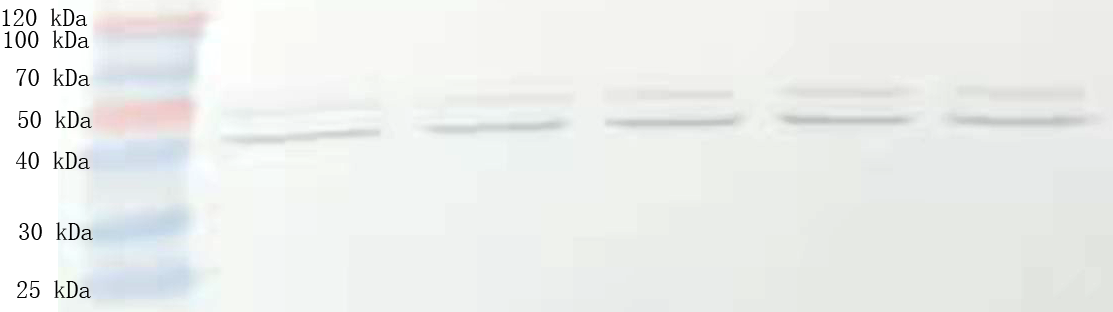
**

**JNK**

**
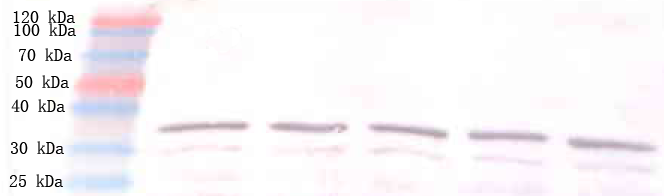
**

**GAPDH**

Supplement: Supplementary file 1 — Additional file 1. [file 12917_2022_3312_MOESM1_ESM.docx]
